# TICAM1-Mediated TLR3/TLR4 Signaling Promotes Endometrial Stromal Cell Proliferation, Migration, and Invasion in Endometriosis via IRF3/IFN-β Axis

**DOI:** 10.3390/ijms27115089

**Published:** 2026-06-04

**Authors:** HaLiSai MuDanLiFu, Suming Huang, Yamei Li, Yan Liang, Xiaoya Zhao, Qian Zhu, Sifan Ji, Jie Zhou, Chuqing He, Shunna Ge, Jian Zhang

**Affiliations:** 1 Department of Obstetrics and Gynecology, The International Peace Maternity and Child Health Hospital, School of Medicine, Shanghai Jiao Tong University, Shanghai 200030, China; 2 Shanghai Municipal Key Clinical Specialty, Shanghai 200030, China; 3 Shanghai Key Laboratory of Embryo Original Diseases, Shanghai 200030, China

**Keywords:** endometriosis, TICAM1, TLR3, TLR4, IRF3, IFN-β, proliferation, migration, invasion

## Abstract

Endometriosis (EMs) is an estrogen-dependent inflammatory disease characterized by the presence of endometrial-like tissue outside the uterine cavity, yet its precise pathogenesis remains incompletely elucidated. TICAM1, a key adaptor protein in the Toll-like receptor (TLR) signaling pathway, is known to be involved in inflammatory responses; however, its specific role in EMs has not been defined. This study integrated evidence from clinical tissue samples of patients with ovarian endometriomas, in vitro studies, and in vivo models to explore the role of TICAM1 in EMs. TICAM1 expression was significantly upregulated in both eutopic and ectopic endometrium, with the highest levels observed in ectopic lesions, where it was primarily localized to stromal and glandular epithelial cells. Functional experiments showed that TICAM1 overexpression promoted the proliferation, migration, and invasion of human endometrial stromal cells (hESCs), while TICAM1 knockdown suppressed these activities. Concurrently, TLR3 and TLR4 were also upregulated in EMs tissues, and their activation increased TICAM1 expression. Knockdown of TICAM1 attenuated the enhanced cellular activities induced by TLR3/TLR4 activation. Mechanistically, IRF3 and IFN-β levels were elevated in both EMs tissues and TICAM1-overexpressing hESCs, while TICAM1 knockdown inhibited TLR3/TLR4-induced IRF3 phosphorylation and subsequent IFN-β production. These findings were further corroborated in a mouse model of EMs. Together, our findings suggest that TICAM1 may enhance the proliferation, migration, and invasion of hESCs by mediating TLR3/TLR4 signaling and promoting IRF3 phosphorylation and subsequent IFN-β production, thereby potentially contributing to EMs progression. Therefore, targeting TICAM1 may represent a potential therapeutic direction for ovarian endometrioma-associated EMs, while its relevance to superficial peritoneal and deep infiltrating EMs requires further investigation.

## 1. Introduction

Endometriosis (EMs) is a chronic, estrogen-dependent inflammatory disorder characterized by the presence of endometrial-like tissue outside the uterine cavity, affecting approximately 6–10% of reproductive-age women [1,2]. As a leading cause of pelvic pain and infertility, EMs severely impairs quality of life and imposes a substantial socioeconomic burden [3,4]. Despite its high prevalence, the pathogenesis of the disease remains elusive, and first-line treatments consisting of hormonal therapy and surgery are limited by high recurrence rates and significant adverse effects [5,6].

The prevailing etiological model is Sampson’s theory of retrograde menstruation, which posits that endometrial fragments reflux into the peritoneal cavity and implant on peritoneal surfaces [7]. However, given that retrograde menstruation occurs in approximately 90% of women without leading to pathology [8,9], disease susceptibility likely depends on intrinsic properties of the endometrium. This concept is central to the “determinant theory of eutopic endometrium,” which postulates that the endometrium in EMs patients is inherently more proliferative, adhesive, and invasive, thereby enabling ectopic lesion development [10]. These pathogenic traits are largely executed by human endometrial stromal cells (hESCs), which exhibit aberrantly heightened proliferation, migration, and invasion in EMs [11,12].

A defining feature of EMs is a sustained inflammatory peritoneal microenvironment, enriched with cytokines, chemokines, and growth factors [13]. Within this milieu, innate immune signaling—particularly through Toll-like receptors (TLRs)—has been implicated in EMs pathophysiology [14,15]. TLRs, such as TLR3 and TLR4, recognize both pathogen-associated molecular patterns (PAMPs) and damage-associated molecular patterns (DAMPs), initiating pro-inflammatory cascades [16] and contributing to sterile inflammation [17,18]. Notably, both receptors are upregulated in the eutopic endometrium of EMs patients, implicating them in disease pathogenesis [19,20].

As a key adaptor protein, TICAM1 (TIR domain-containing adaptor molecule 1, also known as TRIF) is essential for signaling downstream of TLR3 and TLR4 [21]. TICAM1 recruitment leads to the phosphorylation of interferon regulatory factor 3 (IRF3) and induction of type I interferons, primarily IFN-β, constituting the canonical TICAM1–IRF3–IFN-β axis that is essential for antiviral responses [22,23]. Beyond infection, this axis increasingly appears to regulate pathological processes in inflammation and cancer by modulating cell proliferation, migration, and invasion [24,25]. For example, in lipopolysaccharide (LPS)-stimulated macrophages, melatonin regulates TLR4-mediated inflammatory gene expression through both MyD88-dependent and TICAM1-dependent signaling pathways, with TICAM1 serving as the key adaptor in the latter [26]. Furthermore, TICAM1 has been identified as a susceptibility gene in thyroid cancer [27], highlighting its broader role in disease pathogenesis. Nonetheless, the functional and mechanistic contributions of TICAM1 to EMs pathogenesis remain poorly understood.

Here, using clinical specimens, primary hESCs, and a mouse model, we investigated the potential function and mechanism of TICAM1 in EMs. Our data indicate that TICAM1 is overexpressed in EMs tissues and may contribute to the proliferation, migration, and invasion of hESCs by linking TLR3/TLR4 signaling to IRF3 phosphorylation and subsequent IFN-β production. These findings suggest a previously underrecognized molecular axis in EMs pathogenesis and indicate that targeting TICAM1 may represent a potential direction for future therapeutic investigation.

## 2. Results

### 2.1. TICAM1 Is Upregulated in EMs Tissues

**Figure 1:**
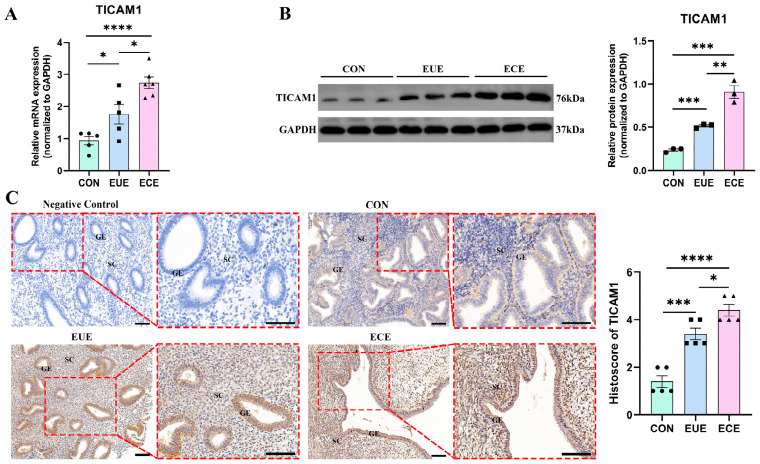
TICAM1 is upregulated in endometrial tissues from patients with endometriosis (EMs). (**A**) RT-qPCR analysis of TICAM1 mRNA expression in CON, EUE, and ECE tissues (*n* = 5–6). (**B**) Representative Western blot images and densitometric quantification of TICAM1 protein expression in CON, EUE, and ECE tissues (*n* = 3). (**C**) Representative immunohistochemical staining images and histoscore analysis of TICAM1 in CON, EUE, and ECE tissues (*n* = 5). Scale bar, 100 µm. Data are presented as mean ± SEM. * *p* < 0.05, ** *p* < 0.01, *** *p* < 0.001, **** *p* < 0.0001. CON, control endometrium; EUE, eutopic endometrium; ECE, ectopic endometrium; GE, glandular epithelium; SC, stromal cell.

TICAM1 expression was initially evaluated in endometrial tissues obtained from the CON, EUE, and ECE groups. RT-qPCR analysis demonstrated that TICAM1 mRNA levels were significantly elevated in the EUE group compared to the CON group, with the highest levels observed in the ECE group (Figure 1A). This upregulation was confirmed at the protein level by Western blot (Figure 1B). Immunohistochemical staining showed a predominant cytoplasmic localization of TICAM1 in both stromal and glandular epithelial cells. Notably, both staining intensity and the proportion of positive cells were markedly higher in the ECE group compared to the EUE and CON groups (Figure 1C). These results indicate that TICAM1 is significantly overexpressed in the pathogenic microenvironment of EMs.

### 2.2. TICAM1 Promotes the Proliferation, Migration, and Invasion of hESCs

**Figure 2:**
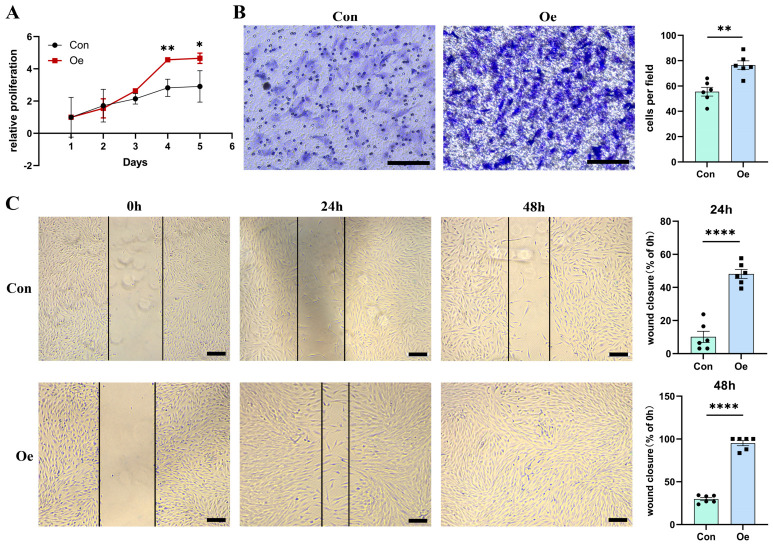
TICAM1 promotes the proliferation, migration, and invasion of human endometrial stromal cells (hESCs). (**A**) Effect of TICAM1 overexpression on hESC proliferation, assessed by CCK-8 assay in Con and Oe groups (*n* = 6). (**B**) Effect of TICAM1 overexpression on hESC invasion, assessed by transwell assay with representative images (scale bar, 100 µm) and quantification of invasive cells (*n* = 6). (**C**) Effect of TICAM1 overexpression on hESC migration, assessed by scratch wound assay with representative images at 24 and 48 h (scale bar, 200 µm) and quantification of relative wound closure rate (*n* = 6). Data are expressed as mean ± SEM. * *p* < 0.05, ** *p* < 0.01, **** *p* < 0.0001. Con, hESCs transfected with an empty plasmid; Oe, hESCs transfected with a TICAM1 overexpression plasmid.

To elucidate the functional implications of TICAM1 upregulation, primary human endometrial stromal cells (hESCs) were isolated (Supplementary Figure S1A,B) and subjected to TICAM1 overexpression (Supplementary Figure S1C). Functional assays demonstrated that TICAM1 overexpression significantly enhanced the proliferative capacity of hESCs, with notable effects observed specifically from day 4 to day 5 (Figure 2A). Moreover, TICAM1 overexpression potentiated the invasive potential of hESCs, as evidenced by a significant enhancement in transwell invasion (Figure 2B) and accelerated wound closure in scratch assays at both 24 and 48 h (Figure 2C). Accordingly, these results suggest that TICAM1 may regulate proliferative, migratory, and invasive cellular phenotypes associated with EMs.

### 2.3. The TLR3/TLR4–TICAM1 Signaling Axis Is Activated in EMs

**Figure 3:**
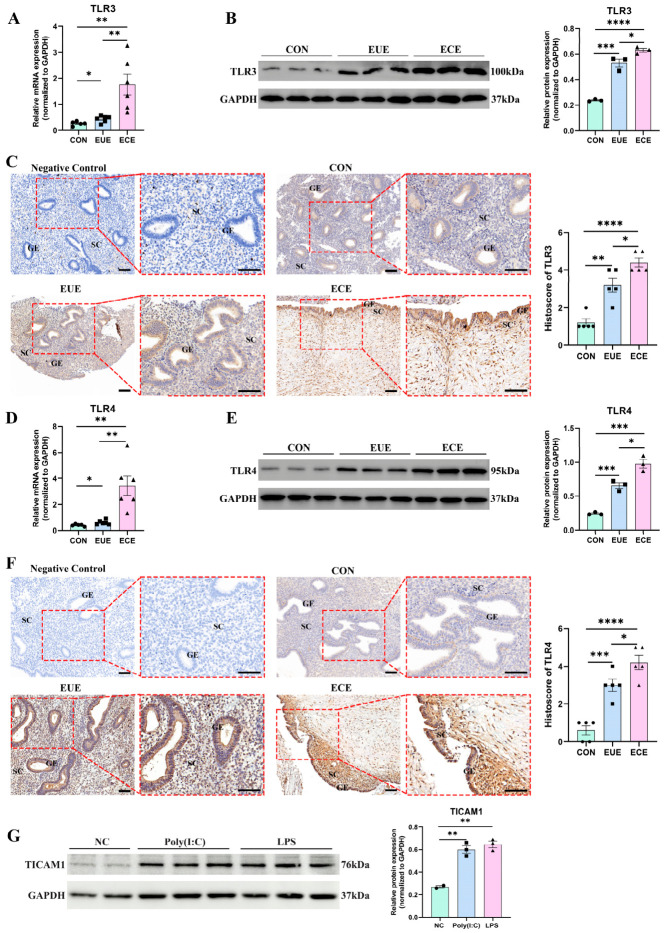
Upregulation of TLR3/TLR4 in EMs and induction of TICAM1. (**A**–**C**) TLR3 expression in endometrial tissues, assessed by RT-qPCR ((**A**); *n* = 5–6), Western blot ((**B**); *n* = 3), and immunohistochemistry with histoscore quantification ((**C**); *n* = 5) in CON, EUE, and ECE tissues. Scale bar, 100 µm. (**D**–**F**) TLR4 expression in endometrial tissues, assessed by RT-qPCR ((**D**); *n* = 5–6), Western blot ((**E**); *n* = 3), and immunohistochemistry with histoscore quantification ((**F**); *n* = 5) in CON, EUE, and ECE tissues. Scale bar, 100 µm. (**G**) Upregulation of TICAM1 protein expression in hESCs following stimulation with the TLR3 agonist Poly(I:C) or the TLR4 agonist LPS for 4 h (*n* = 2–3). Data are presented as mean ± SEM. * *p* < 0.05, ** *p* < 0.01, *** *p* < 0.001, **** *p* < 0.0001. CON, control endometrium; EUE, eutopic endometrium; ECE, ectopic endometrium; GE, glandular epithelium; SC, stromal cell.

Given that TICAM1 serves as the central adaptor for TLR3/TLR4 and is markedly overexpressed in endometriotic tissues (Figure 1), we postulated that the TLR3/TLR4–TICAM1 signaling axis is aberrantly activated, thereby contributing to disease pathogenesis. Indeed, TLR3 and TLR4 mRNA levels were significantly elevated in ECE tissues compared to CON or EUE tissues (Figure 3A,D). Western blot analysis further confirmed these findings, demonstrating that TLR3 and TLR4 protein levels were significantly elevated in both EUE and ECE tissues, with the highest expression observed in the ECE group (Figure 3B,E). Immunohistochemistry (IHC) confirmed their predominant cytoplasmic localization in stromal and epithelial cells, with histoscores being significantly higher in endometriotic tissues (Figure 3C,F). Notably, experimental stimulation of hESCs with the TLR3 agonist Poly(I:C) or the TLR4 agonist LPS increased TICAM1 protein expression (Figure 3G). These findings indicate that the TLR3/TLR4–TICAM1 signaling axis is activated in EMs and suggest that TICAM1 may act as a downstream effector.

### 2.4. TICAM1 Contributes to TLR3/TLR4-Mediated Enhancement of hESC Proliferation, Migration, and Invasion

**Figure 4:**
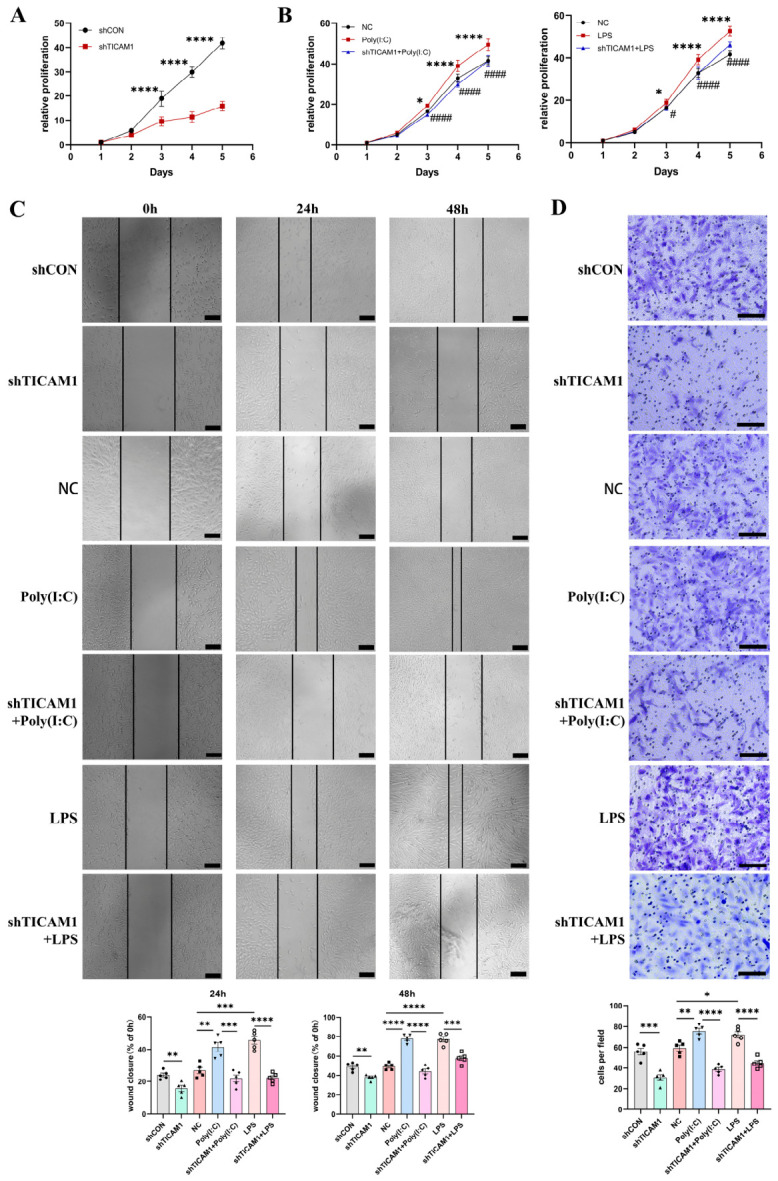
TICAM1 mediates TLR3/TLR4-driven proliferation, migration, and invasion in hESCs. (**A**) Effect of TICAM1 knockdown on hESC proliferation, assessed by CCK-8 assay in shCon and shTICAM1 groups (*n* = 5). (**B**) Effect of TICAM1 knockdown on TLR3/4 agonist-induced hESC proliferation, assessed by CCK-8 assay. Left panel: Cells treated with the TLR3 agonist Poly(I:C). Right panel: Cells treated with the TLR4 agonist LPS. Statistical significance: *, comparison between NC and agonist treatment; #, comparison between agonist treatment alone and agonist treatment with TICAM1 knockdown (*n* = 5). (**C**) Effect of TICAM1 knockdown on hESC migration, assessed by scratch wound assay with representative images at 0, 24, and 48 h (scale bar, 200 µm) and quantification of relative wound closure rate (*n* = 5). (**D**) Effect of TICAM1 knockdown on hESC invasion, assessed by transwell assay with representative images (scale bar, 100 µm) and quantification of invasive cells (*n* = 5). Data are presented as mean ± SEM. * *p* < 0.05, ** *p* < 0.01, *** *p* < 0.001, **** *p* < 0.0001; # *p* < 0.05, #### *p* < 0.0001. shCon, hESCs transfected with control shRNA; shTICAM1, hESCs transfected with TICAM1-specific shRNA; NC, negative control (hESCs treated with PBS); shTICAM1 + Poly(I:C)/LPS, hESCs with TICAM1 knockdown treated with Poly(I:C) or LPS, respectively.

To assess the involvement of TICAM1 in TLR3/TLR4-mediated functional enhancement in hESCs, loss-of-function experiments were conducted using the most efficient shRNA (sh3; Supplementary Figure S1D). TICAM1 depletion significantly reduced basal hESC proliferation (Figure 4A) and attenuated the pro-proliferative effects induced by the TLR3 and TLR4 agonists, Poly(I:C) and LPS (Figure 4B). Similarly, TICAM1 knockdown suppressed both basal and agonist-induced migration in wound healing assays (Figure 4C). Furthermore, transwell invasion assays demonstrated that TICAM1 knockdown inhibited both basal and TLR3/TLR4-stimulated invasion capacity (Figure 4D). Together, these loss-of-function findings suggest that TICAM1 contributes to the proliferative, migratory, and invasive effects of TLR3/TLR4 signaling in hESCs.

### 2.5. TICAM1 Links TLR3/TLR4 Signaling to the IRF3/IFN-β Pathway

**Figure 5:**
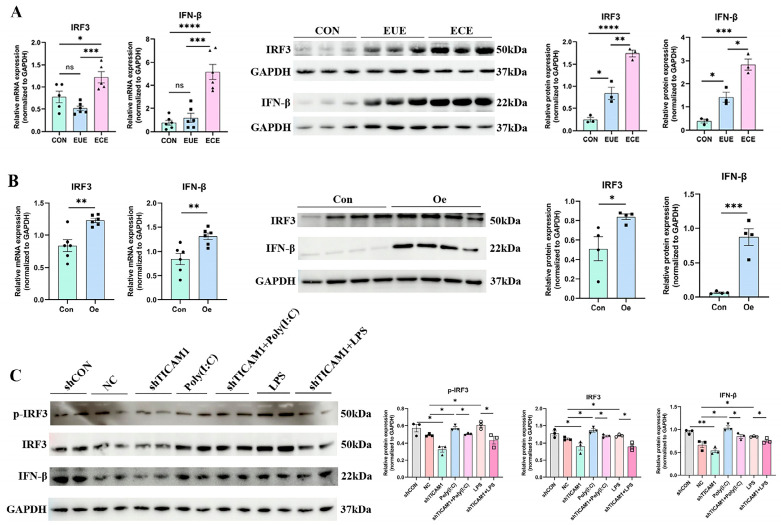
TICAM1 mediates TLR3/TLR4-induced IRF3 phosphorylation and IFN-β expression. (**A**) IRF3 and IFN-β expression in endometrial tissues, assessed by RT-qPCR (left; *n* = 5–6) and Western blot (right; *n* = 3) in CON, EUE, and ECE tissues. (**B**) Effect of TICAM1 overexpression on IRF3 and IFN-β expression in hESCs, assessed by RT-qPCR (left; *n* = 6) and Western blot (right; *n* = 4) in Con and TICAM1-overexpressing (Oe) cells. (**C**) Effect of TICAM1 knockdown on TLR3/TLR4 agonist-induced IRF3 phosphorylation and IFN-β production in hESCs. Representative Western blots and densitometric quantification of p-IRF3, IRF3, and IFN-β in hESCs under the indicated conditions (*n* = 3). Data are presented as mean ± SEM. * *p* < 0.05, ** *p* < 0.01, *** *p* < 0.001, **** *p* < 0.0001; ns, not significant (*p* > 0.05). CON, control endometrium; EUE, eutopic endometrium; ECE, ectopic endometrium; Con, hESCs transfected with empty plasmid; Oe, hESCs transfected with TICAM1 overexpression plasmid; shCon, hESCs transfected with control shRNA; shTICAM1, hESCs transfected with TICAM1-specific shRNA; NC, negative control (hESCs treated with PBS); shTICAM1 + Poly(I:C)/LPS, hESCs with TICAM1 knockdown treated with Poly(I:C) or LPS, respectively.

Having established that TICAM1 mediates TLR3/TLR4 signaling to promote aberrant cellular functions, we next interrogated the downstream signaling pathway. Key components of the canonical TICAM1 pathway, IRF3 and IFN-β, were significantly upregulated at both transcriptional and translational levels in ECE tissues (Figure 5A). Gain- and loss-of-function experiments in hESCs demonstrated that TICAM1 overexpression increased IRF3 and IFN-β expression (Figure 5B). Conversely, TICAM1 knockdown decreased basal levels of phosphorylated IRF3 (p-IRF3), total IRF3, and IFN-β, and blunted their induction upon TLR3 or TLR4 activation (Figure 5C). These results support the conclusion that IRF3 activation and subsequent IFN-β production are functionally downstream of the TLR3/TLR4–TICAM1 axis in EMs.

### 2.6. In Vivo Validation of the TLR3/TLR4–TICAM1–IRF3/IFN-β Axis in a Mouse Model of EMs

**Figure 6:**
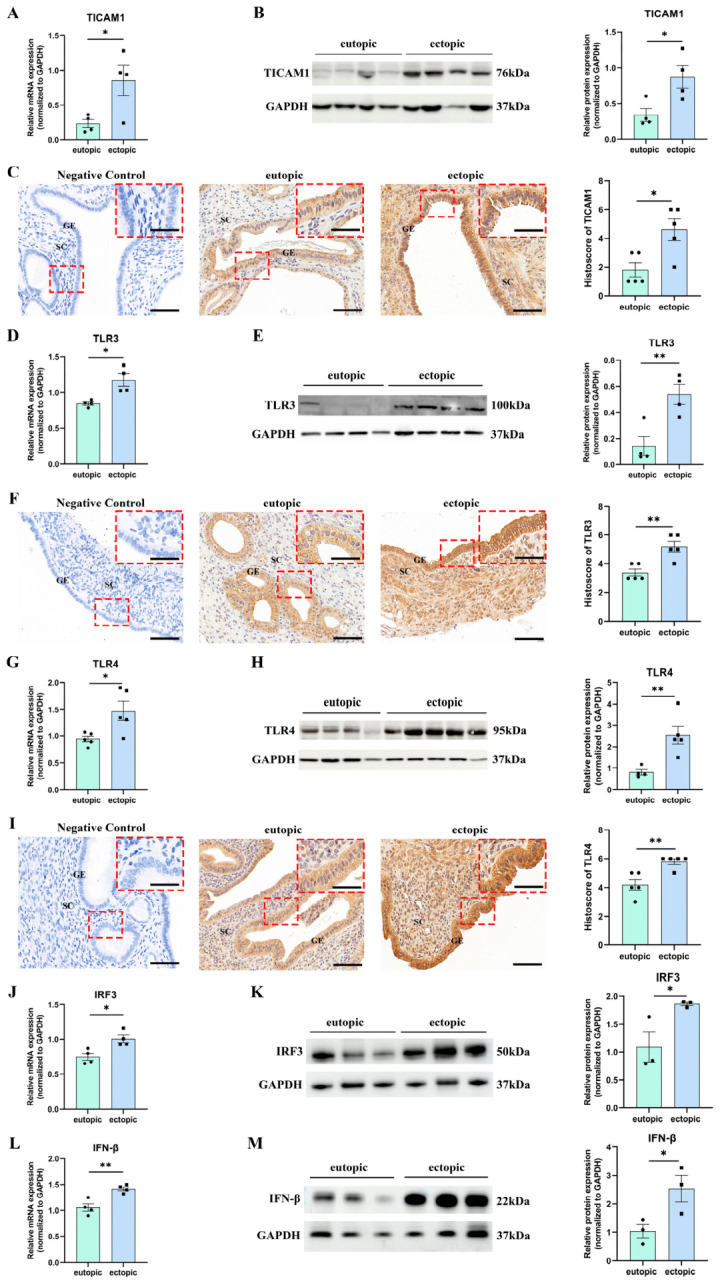
Expression profiles of TICAM1 and related signaling molecules in a mouse model of EMs. Expression levels of target molecules were analyzed in eutopic and ectopic endometrial tissues isolated from estrogen-treated mice (6 weeks). (**A**–**C**) TICAM1 expression, assessed by RT-qPCR ((**A**); *n* = 4), Western blot ((**B**); *n* = 4), and immunohistochemistry ((**C**); *n* = 5). (**D**–**F**) TLR3 expression, assessed by RT-qPCR ((**D**); *n* = 4), Western blot ((**E**); *n* = 4), and immunohistochemistry ((**F**); *n* = 5). (**G**–**I**) TLR4 expression, assessed by RT-qPCR ((**G**); *n* = 5), Western blot ((**H**); *n* = 4–5), and immunohistochemistry ((**I**); *n* = 5). (**J**,**K**) IRF3 expression, assessed by RT-qPCR ((**J**); *n* = 4) and Western blot ((**K**); *n* = 3). (**L**,**M**) IFN-β expression, assessed by RT-qPCR ((**L**); *n* = 4) and Western blot ((**M**); *n* = 3). For immunohistochemistry images (**C**,**F**,**I**), scale bars represent 100 µm for the main images and 50 µm for the insets. Data are presented as mean ± SEM. * *p* < 0.05, ** *p* < 0.01. GE, glandular epithelium; SC, stromal cell.

To validate the in vivo pathophysiological significance of this signaling axis, we generated an EMs mouse model and compared two different estrogen treatment periods (6 weeks versus 8 weeks). This design allowed us to assess the impact of prolonged estrogen exposure on lesion progression and activation of the TLR3/TLR4–TICAM1–IRF3/IFN-β pathway. Successful engraftment of lesions was confirmed (Supplementary Figure S2A–C). Quantitative measurement of excised lesions showed that 8-week estrogen treatment resulted in significantly greater lesion weight and volume compared with the 6-week regimen, whereas the number of established lesions did not differ significantly between the two groups (Supplementary Figure S2D–F). This suggests an estrogen-dependent growth response without altering implantation efficiency. Ectopic lesions in both experimental groups displayed significantly upregulated TICAM1 mRNA expression relative to eutopic endometrium, with a further increase observed after the 8-week estrogen treatment (Figure 6A; Supplementary Figure S2G). Western blot and immunohistochemistry confirmed this upregulation and showed predominant cytoplasmic localization of TICAM1 in stromal and glandular epithelial cells (Figure 6B,C). Regarding upstream signaling, TLR3 and TLR4 transcript levels were markedly elevated in ectopic lesions compared with eutopic endometrium (Figure 6D,G). Notably, lesions from the 6-week estrogen exposure group had elevated mRNA levels of both receptors relative to those from the 8-week group (Supplementary Figure S2H,I). Consistent with these transcriptional profiles, protein expression of TLR3 and TLR4 was increased, displaying predominant cytoplasmic localization in stromal and glandular epithelial cells of ectopic tissues (Figure 6E,F,H,I). Downstream of this pathway, IRF3 and IFN-β expression was markedly upregulated in ectopic lesions relative to eutopic endometrium in the 6-week group (Figure 6J,L). While a similar trend was observed in the 8-week group, the magnitude of IRF3 and IFN-β upregulation in ectopic lesions was significantly more pronounced in the 6-week cohort (Supplementary Figure S2J,K). Western blot analysis further validated the enhanced protein expression of IRF3 and IFN-β in ectopic lesions from the 6-week treatment group (Figure 6K,M).

## 3. Discussion

This study provides evidence that TICAM1 may contribute to EMs pathogenesis by bridging TLR3/TLR4 activation to the IRF3–IFN-β signaling axis in hESCs. We observed a progressive upregulation of TICAM1 from control endometrium (CON) to eutopic (EUE) and ectopic lesions (ECE) at both mRNA and protein levels, with expression predominantly localized to stromal and glandular epithelial cells. This distinct expression pattern suggests a potential association between TICAM1 upregulation and EMs-associated pathological processes. While consistent with a prior report of elevated TICAM1 mRNA in eutopic endometrium [28], our work extends this observation by confirming protein overexpression and evaluating its functional consequences.

The enhanced proliferative, migratory, and invasive capacities of hESCs are fundamental to ectopic lesion establishment [29,30]. Our functional experiments showed that TICAM1 overexpression enhanced these cellular behaviors in primary hESCs, whereas its knockdown suppressed them, providing functional evidence that TICAM1 may contribute to these phenotypes in the context of EMs. Notably, blockade of the TICAM1-dependent pathway has been shown to protect against pancreatic cancer by modulating tumor-associated inflammation [31], reinforcing the view that TICAM1-mediated signaling can contribute to pathogenic inflammation relevant to tumorigenesis. Consistent with this, our study suggests a potential pro-pathogenic role for TICAM1 in EMs, indicating that TICAM1’s pathogenic relevance may extend to a benign gynecological context.

A mechanistic insight is the positioning of TICAM1 within a specific signaling axis. We observed the overexpression of TLR3 and TLR4 in EMs tissues and showed that experimental activation of these receptors with Poly(I:C) and LPS can upregulate TICAM1 expression in hESCs. This suggests a feed-forward mechanism in which TLR3/TLR4 activation induces TICAM1 expression. Such amplification could be particularly relevant in the peritoneal microenvironment, where chronic exposure to endogenous TLR ligands (e.g., HMGB1, extracellular RNA) or microbial components from subclinical infections may contribute to sustained activation of this pathway [32,33]. Consequently, our findings suggest a possible molecular mechanism through which initial infection or sterile inflammation—which have been proposed to contribute to EMs pathogenesis [34]—may be linked to sustained cellular pathogenicity. Wróbel et al. reported significantly altered plasma proteasome levels in patients with mild (Stage I/II) EMs compared to controls, as well as differential plasma immunoproteasome levels between patients with and without ovarian cysts, suggesting a potentially active role of these proteolytic complexes in the pathogenesis of EMs [35]. Kacperczyk-Bartnik et al. further assessed plasma and peritoneal fluid levels of poly(ADP-ribose) polymerase (PARP) in women with and without EMs and found no overall differences between groups, although plasma PARP concentrations were higher in women with a history of infertility [36]. Together, these findings underscore that the inflammatory microenvironment and systemic biochemical milieu in EMs may involve multiple interconnected processes, including TLR-mediated signaling, proteostatic regulation, PARP-related pathways, and immune responses.

Extending these findings, our data further characterize a signaling cascade from TLR3/TLR4 through TICAM1 to IRF3 activation and IFN-β production in hESCs. Our data indicate that TICAM1 contributes to TLR3/TLR4-mediated IRF3 phosphorylation and subsequent IFN-β induction, supporting its role as an adaptor in this context. While this pathway is well-characterized in immune cells [21,37], its presence and functional significance in hESCs, and by extension in EMs pathogenesis, had not been previously elucidated.

In vivo data from a mouse EMs model further support the biological relevance of our findings. Compared with eutopic endometrium, ectopic lesions in both the 6-week and 8-week estrogen treatment groups exhibited elevated expression of TICAM1, TLR3, TLR4, IRF3, and IFN-β. Notably, TICAM1 expression was higher in ectopic lesions from the 8-week group than in those from the 6-week group, whereas TLR3, TLR4, IRF3, and IFN-β expression were more pronounced in ectopic lesions from the 6-week group than in those from the 8-week cohort. This temporal dissociation suggests that chronic estrogen exposure may further upregulate TICAM1 in parallel with lesion progression, whereas the upstream receptors and downstream effectors peak at an earlier stage of lesion development. Chronic estrogen exposure and prolonged lesion growth may induce compensatory anti-inflammatory mechanisms, such as IRAK-M [38], that dampen TLR3/TLR4 and IRF3/IFN-β signaling over time. These findings suggest a dynamic interplay between hormonal and inflammatory pathways in EMs [39,40].

Translationally, our findings suggest that targeting TICAM1 may represent a potential direction for future therapeutic investigation in EMs. As an adaptor protein downstream of TLR3/TLR4, TICAM1 offers theoretical advantages for therapeutic intervention. Unlike direct targeting of TLR3 or TLR4, targeting TICAM1 could more selectively disrupt pathogenic signaling while preserving MyD88-dependent protective functions. Currently, several small-molecule inhibitors targeting the TLR pathway have shown potent therapeutic effects in preclinical models. For instance, the TLR4 antagonist TAK-242, when combined with antibiotics, demonstrated strong efficacy in an E. coli-induced sepsis model using BCG-primed mice [41], providing evidence for targeting this signaling pathway. Building on this, specific TICAM1-interfering peptides (e.g., TIR domain-containing competitive peptides) could be designed in the future to disrupt TICAM1 recruitment to activated receptors. Future studies could evaluate these agents in EMs mouse models to determine whether pharmacological blockade of the TLR3/TLR4–TICAM1 axis reduces lesion burden and pathogenic cellular behaviors, thereby establishing TICAM1 as a potential target.

Several limitations of this study warrant acknowledgment. First, although our loss- and gain-of-function experiments suggest that TICAM1 contributes to TLR3/TLR4-induced IRF3 phosphorylation, we did not provide direct biochemical evidence (e.g., co-immunoprecipitation) to confirm the physical interaction between TICAM1 and the downstream kinases TBK1/IKKε in hESCs. In the canonical TLR3/TLR4 pathway, TICAM1 acts as a scaffold protein to recruit TBK1/IKKε, which subsequently phosphorylate IRF3 [14,42]. Therefore, future studies using co-immunoprecipitation are needed to directly validate these interactions, whereas specific kinase inhibitors could help to establish the functional hierarchy downstream of TICAM1 in EMs. Second, we did not perform classical rescue experiments to functionally validate the observed phenotypes. Specifically, we did not reconstitute IFN-β expression in TICAM1-knockdown hESCs; therefore, we cannot definitively conclude that IFN-β is the direct downstream effector mediating the proliferative and invasive phenotypes. Additionally, although we used three independent shRNAs to screen for the most efficient knockdown construct, we did not perform TICAM1 re-expression or use IRF3 inhibitors or IFN-β neutralizing antibodies. Without these controls, we cannot completely exclude potential contributions from non-specific shRNA effects or parallel signaling pathways (e.g., NF-κB). Future research incorporating these rescue strategies will further strengthen our conclusions. Third, while our mouse model validated the upregulation of the TLR3/TLR4–TICAM1–IRF3/IFN-β axis in ectopic lesions—a molecular alteration highly consistent with the proliferative and invasive phenotypes observed in primary hESCs in vitro—we did not further evaluate functional histological markers of proliferation (e.g., Ki-67, PCNA) or invasion/EMT (e.g., MMP-2/9, vimentin, E-cadherin) [43,44] within the murine tissues. Future studies incorporating multiplex immunofluorescence or spatial transcriptomics, alongside conditional TICAM1 knockout models, will be essential to directly establish the in vivo histopathological correlates of these cellular behaviors and bridge the gap between molecular signaling and pathological progression. Furthermore, our findings are based exclusively on ovarian endometrioma samples. TICAM1 signaling may behave differently in peritoneal or deep infiltrating EMs, which represents an important direction for future investigation. Additionally, although our in vitro models enabled manipulation of TICAM1, they cannot fully recapitulate the complex multicellular peritoneal microenvironment in vivo, and the precise initial triggers for TLR3/TLR4 activation in EMs—whether endogenous DAMPs from retrograde menstruation or microbial components [45,46]—remain incompletely defined. Finally, given the limited sample size of this study, our current findings should be considered exploratory and may help in identifying potential biomarkers and underlying mechanisms. Definitive validation of these biomarkers and confirmation of the proposed mechanism will require future studies with larger, independent cohorts.

Notwithstanding these limitations, our study advances the understanding of EMs pathogenesis by suggesting that TICAM1 may serve as an adaptor linking TLR3/TLR4 signaling to IRF3 phosphorylation and IFN-β production. These insights may help to clarify part of the molecular basis of EMs and support further investigation of this pathway in future therapeutic studies.

## 4. Materials and Methods

### 4.1. Tissue Collection

This study was approved by the Institutional Ethics Committee of the International Peace Maternity and Child Health Hospital (IPMCH; Approval No.: GKLW 2016-42), and written informed consent was obtained from all participants. Endometrial tissues were collected from premenopausal women who underwent surgery at the IPMCH. All participants were 26–47 years of age, had regular menstrual cycles, and had not received any hormonal therapy for at least 3 months prior to surgery. The exclusion criteria were as follows: (1) histological or imaging evidence of adenomyosis; (2) presence of an intrauterine device; (3) positive preoperative vaginal cultures for bacteria, fungi, or mycoplasma; (4) history of pregnancy within the preceding 6 months; and (5) a history of malignancy. The EMs group comprised 16 patients with ovarian endometriomas histopathologically confirmed following ovarian cystectomy. Disease severity was classified according to the revised American Society for Reproductive Medicine (rASRM) classification as follows: stage II (*n* = 1), stage III (*n* = 10), and stage IV (*n* = 5). For each patient in the EMs group, eutopic endometrium (EUE) and ectopic endometrium (ECE) samples were collected during surgery. The control (CON) group comprised 15 patients who underwent surgery for benign gynecological conditions, and their endometrial tissues were obtained during the procedure. These conditions were histopathologically diagnosed as uterine leiomyoma or mature cystic teratoma.

### 4.2. Cell Culture and Treatments

Primary human endometrial stromal cells (hESCs) were obtained from fresh endometrial tissue, as previously described, with some modifications [47]. Briefly, minced tissue was digested with collagenase I (10 mg in 10 mL DMEM/F-12 containing 20 µL DNase I) for 15 min at 37 °C. The resulting cell suspension was filtered through a 40 µm strainer and centrifuged at 1500 rpm for 5 min. The pellet was resuspended in DMEM/F-12 (Gibco, Grand Island, NY, USA) with 20% FBS (Gibco, Grand Island, NY, USA) and plated onto 60 mm dishes. The purity of hESCs was verified by immunofluorescence staining, which showed positive staining for vimentin and negative staining for cytokeratin 7 (Supplementary Figure S1A,B). Cells were maintained in DMEM/F-12 with 10% FBS and 1% penicillin–streptomycin (Gibco, Grand Island, NY, USA) at 37 °C in a humidified incubator with 5% CO_2_, and the medium was refreshed every 48 h. For passaging, cells were rinsed with PBS (Absin, Shanghai, China), trypsinized, collected in complete medium, and centrifuged at 1000 rpm for 4 min. The pellet was resuspended and seeded at a 1:2 ratio into new culture dishes. Early-passage cells (P3–P5) were used for all experiments. TLR activation was performed by treating hESCs with Poly(I:C) (10 µg/mL, TLR3 agonist) or LPS (100 ng/mL, TLR4 agonist) (Selleck, Houston, TX, USA) for 4 h [48]. The LPS concentration was selected based on previous studies using hESCs, which identified this dose as optimal without inducing overt cytotoxicity [49]. TICAM1 expression was modulated by transfection, and cells were cultured for 48 h before TLR agonist stimulation.

### 4.3. Plasmid Construction and Transfection

TICAM1 overexpression plasmid and three specific shRNAs targeting TICAM1 were obtained from You Bio (Changsha, China). Transfection was carried out with jetPRIME^®^ (Polyplus-transfection, Strasbourg, France) following the manufacturer’s protocol. Briefly, cells were plated at 2 × 10^5^ per well in 6-well plates (2 mL medium) one day before transfection, and transfection was performed at 60–80% confluence. For each well, 2 µg DNA was mixed with 200 µL jetPRIME^®^ buffer, then 4 µL jetPRIME^®^ reagent was added after brief vortexing, and the mixture was vortexed again. Following a 10 min incubation at room temperature, the mixture was added dropwise and the plate was gently swirled before being returned to the incubator. After 6 h, the medium was replaced with fresh DMEM/F-12 containing 10% FBS. Cells were further cultured for 48 h before evaluating transgene expression and knockdown efficiency.

### 4.4. RNA Extraction and Quantitative Real-Time PCR (RT-qPCR)

RNA was isolated from tissue and cell samples using Vezol Reagent (Vazyme, Nanjing, China). Tissue specimens were homogenized with grinding beads, whereas adherent cells were directly lysed in 6-well plates. Lysates were subjected to phenol–chloroform extraction with chloroform:isoamyl alcohol (24:1, *v*/*v*; Acmec Biochemical, Shanghai, China), and RNA was precipitated with isopropanol and washed with ethanol. RNA concentration and purity were measured using a NanoDrop spectrophotometer (Thermo Fisher Scientific, Waltham, MA, USA), and an A260/A280 ratio of 1.8–2.0 was considered indicative of high quality. Purified RNA was either stored at −80 °C or immediately reverse-transcribed with HiScript III All-in-One RT SuperMix Perfect for qPCR (Vazyme, Nanjing, China). Reverse transcription was performed at 50 °C for 15 min, followed by 85 °C for 5 s. Quantitative PCR was carried out using ChamQ Universal SYBR qPCR Master Mix (Vazyme, Nanjing, China), following the method of a previously published study with minor modifications [50]. Each 10 µL reaction contained 5 µL 2× Master Mix, 0.2 µL of each primer (10 µM), 4 µL cDNA template, and 0.6 µL nuclease-free water. The thermal cycling program was as follows: initial denaturation at 95 °C for 30 s, followed by 40 cycles at 95 °C for 10 s and 60 °C for 30 s, followed by melting curve analysis. Relative gene expression was calculated using the 2^−ΔΔCt^ method. Primer sequences are listed in Supplementary Tables S1 and S2.

### 4.5. Protein Extraction and Western Blot Analysis

Protein extraction and Western blot analysis were performed according to previously published protocols with minor modifications [49]. Proteins were isolated from tissue and cell samples using RIPA lysis buffer (Epizyme, Shanghai, China) containing protease and phosphatase inhibitors. Following homogenization, the lysates were centrifuged to remove debris, and the supernatant was collected. Protein concentration was determined using a BCA assay. Equal protein aliquots were resolved on 10% SDS-PAGE gels and transferred onto PVDF membranes (Millipore, Merck KGaA, Darmstadt, Germany). Membranes were blocked with 5% skim milk in TBST at room temperature for 1 h, then incubated overnight at 4 °C with primary antibodies directed against TICAM1 (1:1000; Proteintech, Wuhan, China), TLR3 (1:1000; Affinity, Changzhou, China), TLR4 (1:2000; Proteintech, Wuhan, China), IRF3 (1:1000; STARTER, Hangzhou, China), p-IRF3 (1:1000; Univ, Shanghai, China), IFN-β (1:5000; Genuinebiotech, Pingdingshan, China), and GAPDH (1:10,000; Proteintech, Wuhan, China). Following TBST washes, membranes were incubated for 1 h at room temperature with HRP-conjugated secondary antibodies (1:10,000; Proteintech, Wuhan, China). Protein signals were visualized using an ECL kit (Epizyme, Shanghai, China) and captured on a chemiluminescence imaging system (Cytiva, Marlborough, MA, USA). Band intensities were quantified using ImageJ software version 1.41o (National Institutes of Health, Bethesda, MD, USA) and normalized to GAPDH as the loading control.

### 4.6. Immunohistochemistry (IHC)

Endometrial tissues were fixed in 4% paraformaldehyde, paraffin-embedded, and sectioned at 4 µm. Sections were deparaffinized in xylene (2 × 15 min), rehydrated through a graded ethanol series (100% × 2, 85%, 75%; 5 min each), and rinsed in distilled water (3 × 5 min). Antigen retrieval was performed in EDTA buffer (diluted in ddH_2_O) using a microwave. The buffer was preheated to boiling at medium power. Slides were then immersed and incubated with the microwave off for 8 min, followed by reheating at medium-low power for 7 min, avoiding drying. After cooling naturally for 3 h, slides were washed with PBS (3 × 5 min). Endogenous peroxidase was blocked with 3% H_2_O_2_ (25 min, dark), and nonspecific binding was blocked with 3% BSA (30 min). Sections were incubated with primary antibodies (anti-TICAM1 1:250, anti-TLR3 1:100, anti-TLR4 1:200) overnight at 4 °C, followed by HRP-conjugated secondary antibodies (50 min). Staining was developed with DAB (Recordbio, Shanghai, China) according to the manufacturer’s protocol, and stopped by rinsing with tap water. Nuclei were counterstained with hematoxylin (90 s), differentiated in acid alcohol (Beyotime, Shanghai, China) (10 s), and blued in PBS (5 min). Sections were dehydrated in ethanol (2 × 5 min), cleared in xylene (2 × 5 min), mounted, and imaged. Immunostaining was scored semiquantitatively by combining the percentage of positive cells and staining intensity. Positive cell percentages were scored as 0 (≤5%), 1 (6–25%), 2 (26–50%), 3 (51–75%), and 4 (>75%). Staining intensity was graded using IHC Profiler: 0 (Negative), 1 (Low Positive), 2 (Positive), 3 (High Positive). The final score (0–7) was the sum of these two metrics.

### 4.7. Immunofluorescence Staining

hESCs were plated at 5 × 10^4^ cells/well in 12-well plates and grown to subconfluence. Immunofluorescence staining was performed with slight adaptations [51]. Cells were fixed with 4% paraformaldehyde (15 min), permeabilized with 0.2% Triton X-100 (10 min), and blocked with 5% normal goat serum (30–60 min). They were then incubated overnight at 4 °C with primary antibodies: anti-vimentin (1:200) and anti-cytokeratin 7 (1:200) (Abcam, Cambridge, UK). After PBS washing, cells were incubated for 1 h in the dark with Alexa Fluor 488-conjugated goat anti-mouse and Alexa Fluor 594-conjugated goat anti-rabbit secondary antibodies (both 1:500; Abcam, Cambridge, UK). Nuclei were stained with DAPI (Yeasen, Shanghai, China; 5–10 min). Images were acquired on a fluorescence microscope and analyzed using ImageJ software version 1.41o (National Institutes of Health, Bethesda, MD, USA) to assess protein expression and localization.

### 4.8. Cell Proliferation Assay

Cell proliferation was assessed using the Cell Counting Kit-8 (CCK-8; Yeasen, Shanghai, China) according to the manufacturer’s instructions. hESCs were transfected with plasmids for TICAM1 overexpression, shRNAs targeting TICAM1, or the corresponding control vectors. Forty-eight hours after transfection, cells were trypsinized, resuspended, and plated in 96-well plates at 3000 cells per well in 100 µL of culture medium. The edge wells of each plate were filled with sterile PBS to reduce edge effects. After cell attachment, Poly(I:C) (10 µg/mL), LPS (100 ng/mL), or PBS vehicle control (negative control, NC) was added to the wells. CCK-8 reagent was added at 0, 24, 48, 72, and 96 h. Following a 2 h incubation at 37 °C, absorbance was read at 450 nm on a microplate reader (Agilent, Santa Clara, CA, USA). Absorbance values were corrected against the blank control at each time point and used to generate a temporal proliferation curve.

### 4.9. Wound Healing Assay

The wound healing assay was performed according to previously reported methods with minor modifications [52]. hESCs were transfected with plasmids for TICAM1 overexpression, shRNAs targeting TICAM1, or the corresponding control vectors. Forty-eight hours after transfection, cells were trypsinized, resuspended at 5 × 10^5^ cells/mL, and plated in 6-well plates (2 mL per well). Reference lines were drawn on the underside of each plate to ensure consistent imaging fields. Once cells reached 80–90% confluence, a linear scratch was created across the monolayer using a 200 µL pipette tip, and debris was removed by washing with PBS. Immediately after wounding, cells were cultured in medium containing Poly(I:C) (10 µg/mL), LPS (100 ng/mL), or PBS vehicle control (negative control, NC). Wound images were captured under a microscope at 0, 24, and 48 h. Wound closure (%) was calculated as: [(Width at 0 h − Width at the given time point)/Width at 0 h] × 100%.

### 4.10. Transwell Invasion Assay

Transwell invasion assays were performed according to previously reported methods with slight modifications [53]. Matrigel (Corning Inc., Corning, NY, USA) was thawed overnight at 4 °C and diluted 1:5 in serum-free DMEM/F-12 medium. A 30 µL aliquot of the diluted Matrigel was added to the upper chamber of each transwell insert and allowed to polymerize in the incubator. Cells were loaded into the upper chamber at 2.5 × 10^5^ cells/mL (200 µL per well), while the lower chamber contained 500 µL of complete medium with 15% FBS as a chemoattractant. After 48 h of incubation, inserts were fixed in 4% paraformaldehyde for 15 min and stained with 0.5% crystal violet for 30 min. Invaded cells on the lower surface were observed under an inverted microscope. Images were captured, and cells in five randomly selected fields per insert were quantified for statistical analysis.

### 4.11. Mouse Model of EMs

A total of 30 female C57BL/6 mice (6–8 weeks old, 18–20 g) were obtained from Gem-Pharmatech Co., Ltd. (Nanjing, China) and maintained in the SPF animal facility of the Experimental Animal Center of IPMCH. Mice were housed under a 12 h light/dark cycle with controlled environmental conditions. Sterilized food and water were provided ad libitum, and cage bedding was replaced regularly to ensure hygiene. Donor mice were acclimated for 1 week and then administered subcutaneous injections of estradiol benzoate (9.75 µL in 150 µL corn oil; CEN’S, Hong Kong, China) on days 1 and 3 of the second week. On day 5, donor mice were euthanized and uteri were excised, minced into 1 mm^3^ fragments, and stored in PBS. Recipient mice were anesthetized via intraperitoneal injection of Avertin (150–300 µL). Approximately 25–30 uterine tissue fragments were injected proximal to the vaginal opening, followed by gentle abdominal massage to facilitate tissue dispersion. Subsequently, recipient mice received supplemental estradiol benzoate injections: 100 µL intramuscularly in the thigh and 150 µL subcutaneously in the head-neck region. Mice were randomly assigned to 6- or 8-week experimental groups and maintained on a regimen of regular estradiol benzoate injections. At the designated endpoints (6 or 8 weeks post-transplantation), mice were euthanized. The endometriotic lesions were harvested from the abdominal cavity. The number, location, and total volume or weight of the lesions were recorded to assess lesion burden. The animal experimental protocol was approved by the Animal Welfare and Ethics Committee of IPMCH (Ethics No.: GKDW-A-2024-13).

### 4.12. Statistical Analysis

Quantitative data are presented as mean ± SEM. The *n* values represent independent biological replicates and are provided in the corresponding figure legends. Normality of distribution was assessed using the Shapiro–Wilk test. When the Shapiro–Wilk test indicated non-normal distribution or when the sample size was too small to reliably assess normality, non-parametric tests were applied. Specifically, differences between two independent groups were analyzed using the Mann–Whitney U test, and comparisons among three or more groups were performed using the Kruskal–Wallis test followed by Dunn’s post hoc test. For data that satisfied the normality assumption (Shapiro–Wilk test, *p* > 0.05), one-way ANOVA followed by Tukey’s post hoc test was used when homogeneity of variances was confirmed (Levene’s test, *p* > 0.05); otherwise, Welch’s ANOVA with Games–Howell post hoc test was applied. For comparisons between two independent groups with normal distribution, the unpaired two-tailed Student’s *t*-test was used when variances were equal (Levene’s test, *p* > 0.05), and Welch’s *t*-test was applied when variances were unequal. All statistical analyses were performed using GraphPad Prism 9.0 (GraphPad Software Inc., San Diego, CA, USA). A *p*-value < 0.05 was considered statistically significant. All reported *p*-values are two-tailed.

## 5. Conclusions

TICAM1 was upregulated in the analyzed EMs tissues and may contribute to the proliferation, migration, and invasion of hESCs. As an adaptor of TLR3/TLR4 signaling, TICAM1 may promote IRF3 phosphorylation and subsequent IFN-β production, thereby contributing to these cellular phenotypes. Together, these findings suggest a role for TICAM1 in the pathogenesis of ovarian endometrioma-associated EMs and indicate that TICAM1-mediated signaling could represent a potential pathway for future therapeutic investigation in this phenotype, whereas its relevance to superficial peritoneal and deep infiltrating EMs requires further investigation.

## Data Availability

The original contributions presented in this study are included in the article/Supplementary Material. Further inquiries can be directed to the corresponding author.

## Supplementary Materials

The following supporting information can be downloaded at: https://www.mdpi.com/article/10.3390/ijms27115089/s1.

## Abbreviations

The following abbreviations are used in this manuscript:

cDNA	complementary DNA
CCK-8	Cell Counting Kit-8
CON	control endometrium
ddH_2_O	double-distilled water
ECE	ectopic endometrium
EMs	endometriosis
EUE	eutopic endometrium
FBS	fetal bovine serum
GAPDH	glyceraldehyde-3-phosphate dehydrogenase
GE	glandular epithelium
hESCs	human endometrial stromal cells
IF	immunofluorescence
IFN-β	interferon-β
IHC	immunohistochemistry
IRF3	interferon regulatory factor 3
LPS	lipopolysaccharide
mRNA	messenger RNA
OD	optical density
Poly(I:C)	polyinosinic-polycytidylic acid
rpm	revolutions per minute
RT-qPCR	real-time quantitative PCR
SC	stromal cell
shRNA	short hairpin RNA
TICAM1	Toll-like receptor adapter molecule 1
TLR3	Toll-like receptor 3
TLR4	Toll-like receptor 4
TRIF	TIR-domain-containing adapter-inducing interferon-β

## References

[1] Carbone G., Nelson K., Baumgartner C., Bode A.M., Takahashi A., Chefetz I. (2023). Endometriosis: Cell Death and Cell Signaling Machinery. Endocrinology.

[2] Horne A.W., Missmer S.A. (2022). Pathophysiology, Diagnosis, and Management of Endometriosis. BMJ.

[3] Giudice L.C., Kao L.C. (2004). Endometriosis. Lancet.

[4] Chen L.-C., Hsu J.-W., Huang K.-L., Bai Y.-M., Su T.-P., Li C.-T., Yang A.C., Chang W.-H., Chen T.-J., Tsai S.-J. (2016). Risk of Developing Major Depression and Anxiety Disorders among Women with Endometriosis: A Longitudinal Follow-up Study. J. Affect Disord..

[5] Bulun S.E., Yilmaz B.D., Sison C., Miyazaki K., Bernardi L., Liu S., Kohlmeier A., Yin P., Milad M., Wei J. (2019). Endometriosis. Endocr. Rev..

[6] Vercellini P., Viganò P., Somigliana E., Fedele L. (2014). Endometriosis: Pathogenesis and Treatment. Nat. Rev. Endocrinol..

[7] Sampson J.A. (1927). Metastatic or Embolic Endometriosis, Due to the Menstrual Dissemination of Endometrial Tissue into the Venous Circulation. Am. J. Pathol..

[8] Halme J., Hammond M.G., Hulka J.F., Raj S.G., Talbert L.M. (1984). Retrograde Menstruation in Healthy Women and in Patients with Endometriosis. Obstet. Gynecol..

[9] Chapron C., Marcellin L., Borghese B., Santulli P. (2019). Rethinking Mechanisms, Diagnosis and Management of Endometriosis. Nat. Rev. Endocrinol..

[10] Lang J. (2010). Promotion and Enhancement of Research on Endometriosis. Zhonghua Fu Chan Ke Za Zhi.

[11] Saunders P.T.K., Horne A.W. (2021). Endometriosis: Etiology, Pathobiology, and Therapeutic Prospects. Cell.

[12] Wei Y., Tan H., Yang R., Yang F., Liu D., Huang B., OuYang L., Lei S., Wang Z., Jiang S. (2023). Gut Dysbiosis-Derived β-Glucuronidase Promotes the Development of Endometriosis. Fertil. Steril..

[13] Jørgensen H., Fedorcsak P., Isaacson K., Tevonian E., Xiao A., Beste M., Qvigstad E., Lauffenburger D., Griffith L. (2022). Endometrial Cytokines in Patients with and without Endometriosis Evaluated for Infertility. Fertil. Steril..

[14] Takeda K., Akira S. (2004). TLR Signaling Pathways. Semin Immunol..

[15] Sobstyl A., Mertowska P., Mertowski S., Tarkowski R., Dudziński D., Kotowski M., Bojarski K., Stelmach B., Chermuła B., Brązert M. (2025). Expression of Toll-like Receptors on Lymphocyte Subpopulations and Their Soluble Forms in Serum and Urine of Women with Endometriosis. Cells.

[16] Imler J.L., Hoffmann J.A. (2001). Toll Receptors in Innate Immunity. Trends Cell Biol..

[17] Matsumoto M., Seya T. (2008). TLR3: Interferon Induction by Double-Stranded RNA Including Poly(I:C). Adv. Drug Deliv. Rev..

[18] Oshiumi H., Sasai M., Shida K., Fujita T., Matsumoto M., Seya T. (2003). TIR-Containing Adapter Molecule (TICAM)-2, a Bridging Adapter Recruiting to Toll-like Receptor 4 TICAM-1 That Induces Interferon-Beta. J. Biol. Chem..

[19] Jiang C., Liu C., Guo J., Chen L., Luo N., Qu X., Yang W., Ren Q., Cheng Z. (2017). The Expression of Toll-like Receptors in Eutopic and Ectopic Endometrium and Its Implication in the Inflammatory Pathogenesis of Adenomyosis. Sci. Rep..

[20] Koval H., Chopiak V., Kamyshnyi A. (2015). mRNA TLR2 and TLR4 expression in the endometrium tissue in women with endometriosis assosiated with infertility. Georgian Med. News.

[21] Yamamoto M., Sato S., Hemmi H., Hoshino K., Kaisho T., Sanjo H., Takeuchi O., Sugiyama M., Okabe M., Takeda K. (2003). Role of Adaptor TRIF in the MyD88-Independent Toll-like Receptor Signaling Pathway. Science.

[22] Hoebe K., Du X., Georgel P., Janssen E., Tabeta K., Kim S.O., Goode J., Lin P., Mann N., Mudd S. (2003). Identification of Lps2 as a Key Transducer of MyD88-Independent TIR Signalling. Nature.

[23] Funami K., Matsumoto M., Obuse C., Seya T. (2016). 14-3-3-Zeta Participates in TLR3-Mediated TICAM-1 Signal-Platform Formation. Mol. Immunol..

[24] Kutikhin A.G. (2011). Association of Polymorphisms in TLR Genes and in Genes of the Toll-like Receptor Signaling Pathway with Cancer Risk. Hum. Immunol..

[25] Bora G., Yaba A. (2021). The Role of Mitogen-Activated Protein Kinase Signaling Pathway in Endometriosis. J. Obstet. Gynaecol. Res..

[26] Xia M.-Z., Liang Y.-L., Wang H., Chen X., Huang Y.-Y., Zhang Z.-H., Chen Y.-H., Zhang C., Zhao M., Xu D.-X. (2012). Melatonin Modulates TLR4-Mediated Inflammatory Genes through MyD88- and TRIF-Dependent Signaling Pathways in Lipopolysaccharide-Stimulated RAW264.7 Cells. J. Pineal Res..

[27] Sigurdson A.J., Brenner A.V., Roach J.A., Goudeva L., Müller J.A., Nerlich K., Reiners C., Schwab R., Pfeiffer L., Waldenberger M. (2016). Selected Single-Nucleotide Polymorphisms in FOXE1, SERPINA5, FTO, EVPL, TICAM1 and SCARB1 Are Associated with Papillary and Follicular Thyroid Cancer Risk: Replication Study in a German Population. Carcinogenesis.

[28] Almasi M.Z., Hosseini E., Jafari R., Aflatoonian K., Aghajanpour S., Ramazanali F., Moini A., Shahhoseini M., Afsharian P., Aflatoonian R. (2021). Evaluation of Toll-like Receptor 3 (TLR3) Signaling Pathway Genes and Its Genetic Polymorphisms in Ectopic and Eutopic Endometrium of Women with Endometriosis. J. Gynecol. Obstet. Hum. Reprod..

[29] Neto J.S., Kho R.M., dos Santos Siufi D.F., Baracat E.C., Anderson K.S., Abrão M.S. (2014). Cellular, Histologic, and Molecular Changes Associated with Endometriosis and Ovarian Cancer. J. Minim. Invasive Gynecol..

[30] Blanco L.P., Salmeri N., Temkin S.M., Shanmugam V.K., Stratton P. (2025). Endometriosis and Autoimmunity. Autoimmun. Rev..

[31] Ochi A., Nguyen A.H., Bedrosian A.S., Mushlin H.M., Zarbakhsh S., Barilla R., Zambirinis C.P., Fallon N.C., Rehman A., Pylayeva-Gupta Y. (2012). MyD88 Inhibition Amplifies Dendritic Cell Capacity to Promote Pancreatic Carcinogenesis via Th2 Cells. J. Exp. Med..

[32] Gong T., Liu L., Jiang W., Zhou R. (2020). DAMP-Sensing Receptors in Sterile Inflammation and Inflammatory Diseases. Nat. Rev. Immunol..

[33] Guo B., Chen J.H., Zhang J.H., Fang Y., Liu X.J., Zhang J., Zhu H.Q., Zhan L. (2023). Pattern-Recognition Receptors in Endometriosis: A Narrative Review. Front. Immunol..

[34] Kobayashi H., Higashiura Y., Shigetomi H., Kajihara H. (2014). Pathogenesis of Endometriosis: The Role of Initial Infection and Subsequent Sterile Inflammation (Review). Mol. Med. Rep..

[35] Wróbel M., Zuzanna Z., Ołdak Ł., Kalicka A., Mańka G., Kiecka M., Spaczyński R.Z., Piekarski P., Banaszewska B., Jakimiuk A. (2023). Evaluation of Proteasome and Immunoproteasome Levels in Plasma and Peritoneal Fluid in Patients with Endometriosis. Int. J. Mol. Sci..

[36] Kacperczyk-Bartnik J., Bartnik P., Goławski K., Sierdziński J., Mańka G., Kiecka M., Lipa M., Warzecha D., Spaczyński R., Piekarski P. (2022). Plasma and Peritoneal Poly (ADP-Ribose) Polymerase Levels in Patients with Endometriosis. Biomedicines.

[37] Nakajima Y., Nishino H., Takahashi K., Nugroho A.E., Hirasawa Y., Kaneda T., Morita H. (2025). Azamollugin, a Mollugin Derivative, Has Inhibitory Activity on MyD88- and TRIF-Dependent Pathways. J. Nat. Med..

[38] Kobayashi K., Hernandez L.D., Galán J.E., Janeway C.A., Medzhitov R., Flavell R.A. (2002). IRAK-M Is a Negative Regulator of Toll-like Receptor Signaling. Cell.

[39] Eisenberg V.H., Zolti M., Soriano D. (2012). Is There an Association between Autoimmunity and Endometriosis?. Autoimmun. Rev..

[40] Lamceva J., Uljanovs R., Strumfa I. (2023). The Main Theories on the Pathogenesis of Endometriosis. Int. J. Mol. Sci..

[41] Takashima K., Matsunaga N., Yoshimatsu M., Hazeki K., Kaisho T., Uekata M., Hazeki O., Akira S., Iizawa Y., Ii M. (2009). Analysis of Binding Site for the Novel Small-Molecule TLR4 Signal Transduction Inhibitor TAK-242 and Its Therapeutic Effect on Mouse Sepsis Model. Br. J. Pharmacol..

[42] Lim K.-H., Staudt L.M. (2013). Toll-like Receptor Signaling. Cold Spring Harb. Perspect. Biol..

[43] Lu J., Kornmann M., Traub B. (2023). Role of Epithelial to Mesenchymal Transition in Colorectal Cancer. Int. J. Mol. Sci..

[44] Shoari A., Ashja Ardalan A., Dimesa A.M., Coban M.A. (2024). Targeting Invasion: The Role of MMP-2 and MMP-9 Inhibition in Colorectal Cancer Therapy. Biomolecules.

[45] Shu X., Xie Y., Shu M., Ou X., Yang J., Wu Z., Zhang X., Zhang J., Zeng H., Shao L. (2024). Acute Effects of TLR3 Agonist Poly(I:C) on Bone Marrow Hematopoietic Progenitor Cells in Mice. Immunol. Lett..

[46] Ciesielska A., Matyjek M., Kwiatkowska K. (2021). TLR4 and CD14 Trafficking and Its Influence on LPS-Induced pro-Inflammatory Signaling. Cell Mol. Life Sci..

[47] Jividen K., Movassagh M.J., Jazaeri A., Li H. (2014). Two Methods for Establishing Primary Human Endometrial Stromal Cells from Hysterectomy Specimens. J. Vis. Exp..

[48] Matsuzaki S., Gremeau A.-S., Pouly J.-L. (2020). Impaired Pathogen-Induced Autophagy and Increased IL-1β and TNFα Release in Response to Pathogenic Triggers in Secretory Phase Endometrial Stromal Cells of Endometriosis Patients. Reprod. BioMed Online.

[49] Zhong Q., Jin Z., Ma J., Liao Z., Lai H., Chen S. (2025). 1,25-Dihydroxy Vitamin D3 Inhibits LPS-Mediated Inflammatory Responses in Endometriosis. Ann. Med..

[50] Liu Y., Lu T., Liu Z., Ning W., Li S., Chen Y., Ge X., Guo C., Zheng Y., Wei X. (2022). Six Macrophage-Associated Genes in Synovium Constitute a Novel Diagnostic Signature for Osteoarthritis. Front. Immunol..

[51] Yan H., Zang R., Cui T., Liu Y., Zhang B., Zhao L., Li H., Zhou J., Wang H., Zeng Q. (2024). PROTAC-Mediated Vimentin Degradation Promotes Terminal Erythroid Differentiation of Pluripotent Stem Cells. Stem Cell Res. Ther..

[52] Wang N., Xu X., Guan F., Zheng Y., Shou Y., Xu T., Shen G., Chen H., Lin Y., Cong W. (2024). α-Catenin Promotes Dermal Fibroblasts Proliferation and Migration during Wound Healing via FAK/YAP Activation. FASEB J..

[53] Laomethakorn P., Tayeh M., Samosorn S., Tananyuthawongse C., Watanapokasin R. (2023). 13-Butoxyberberine Bromide Inhibits Migration and Invasion in Skin Cancer A431 Cells. Molecules.

