# Supplementary material for: TICAM1-Mediated TLR3/TLR4 Signaling Promotes Endometrial Stromal Cell Proliferation, Migration, and Invasion in Endometriosis via IRF3/IFN-β Axis
Source: Int J Mol Sci. 2026 Jun 4;27(11):5089. doi: 10.3390/ijms27115089 (PMC13257043; doi:10.3390/ijms27115089)
Supplement: Supplementary file 1 [file ijms-27-05089-s001.zip › ijms-4311285-supplementary.pdf]

International Journal of *Molecular Sciences*

Supporting Information

TICAM1-mediated TLR3/TLR4 signaling promotes endometrial stromal cell proliferation, migration, and invasion in endometriosis via IRF3/IFN-β axis

HaLiSai MuDanLiFu[1,2,3], Suming Huang[1,2,3], Yamei Li[1,2,3], Yan Liang[1,2,3], Xiaoya Zhao[1,2,3], Qian Zhu[1,2,3], Sifan Ji[1,2,3], Jie Zhou[1,2,3], Chuqing He[1,2,3], Shunna Ge[1,2,3,*], Jian Zhang[1,2,3,*]

1 Department of Obstetrics and Gynecology, International Peace Maternity and Child Health Hospital, School of Medicine, Shanghai Jiao Tong University, Shanghai, China
2 Shanghai Municipal Key Clinical Specialty, Shanghai, China
3 Shanghai Key Laboratory of Embryo Original Diseases, Shanghai, China
* Correspondence: geshunna@shsmu.edu.cn (Shunna Ge); zhangjian_ipmch@sjtu.edu.cn (Jian Zhang))

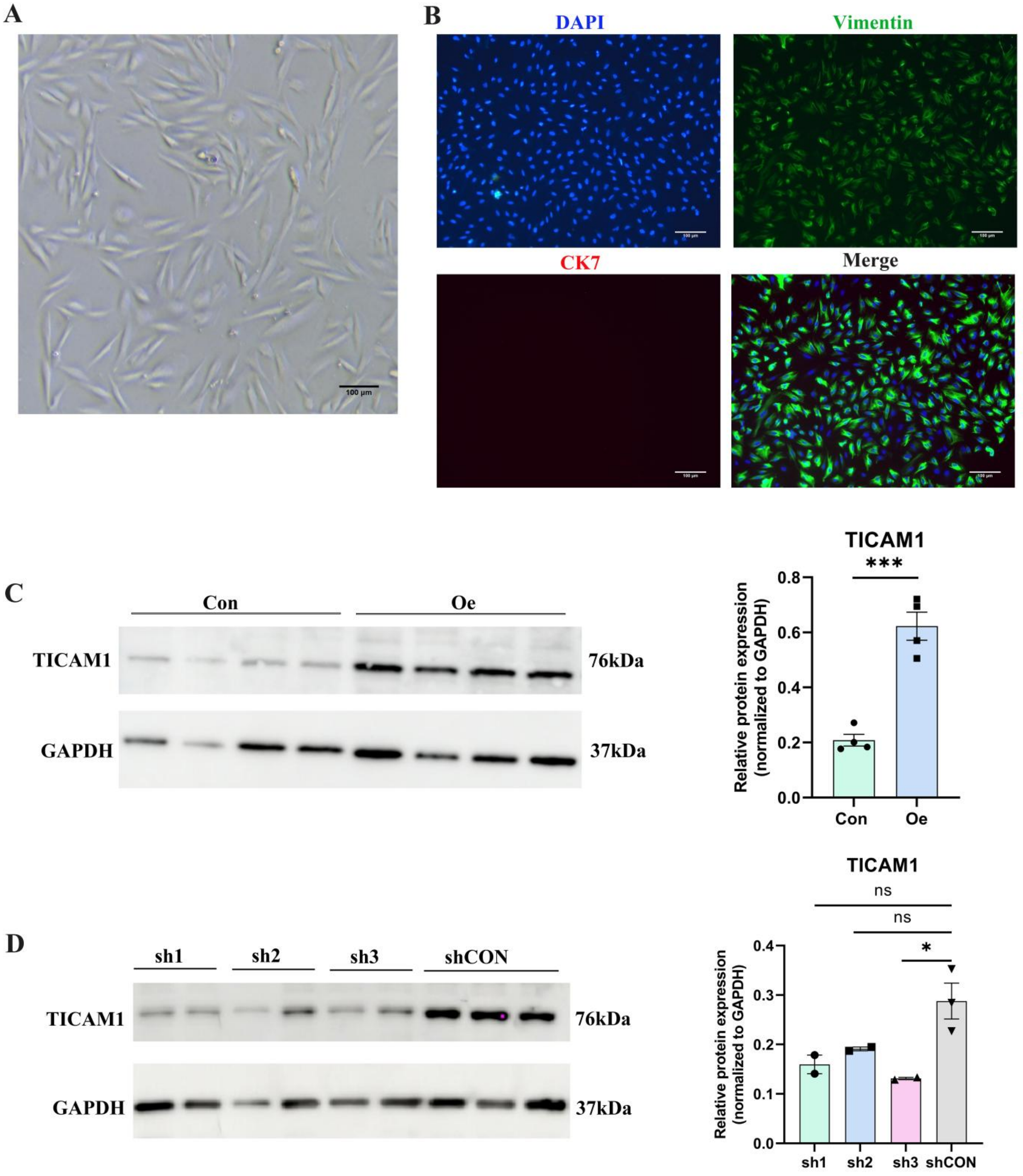

Supplementary Figure S1. Identification and TICAM1 modulation of primary human endometrial stromal cells (hESCs). (**A**) Phase-contrast image of hESCs. Scale bar, 100 μm. (**B**) Immunofluorescence staining of hESCs for the stromal marker vimentin (green) and the epithelial marker cytokeratin 7 (CK7, red). Scale bar, 100 μm. (**C**) Western blot analysis and densitometric quantification confirmed TICAM1 protein overexpression (n = 4). (**D**) TICAM1 knockdown efficiency validated by Western blot and densitometric quantification using three independent shRNAs (n = 2–3). Data are presented as mean ± SEM. * $p < 0.05$, *** $p < 0.001$; ns, not significant ($p > 0.05$). Con, hESCs transfected with empty plasmid; Oe, hESCs transfected with TICAM1 overexpression plasmid.

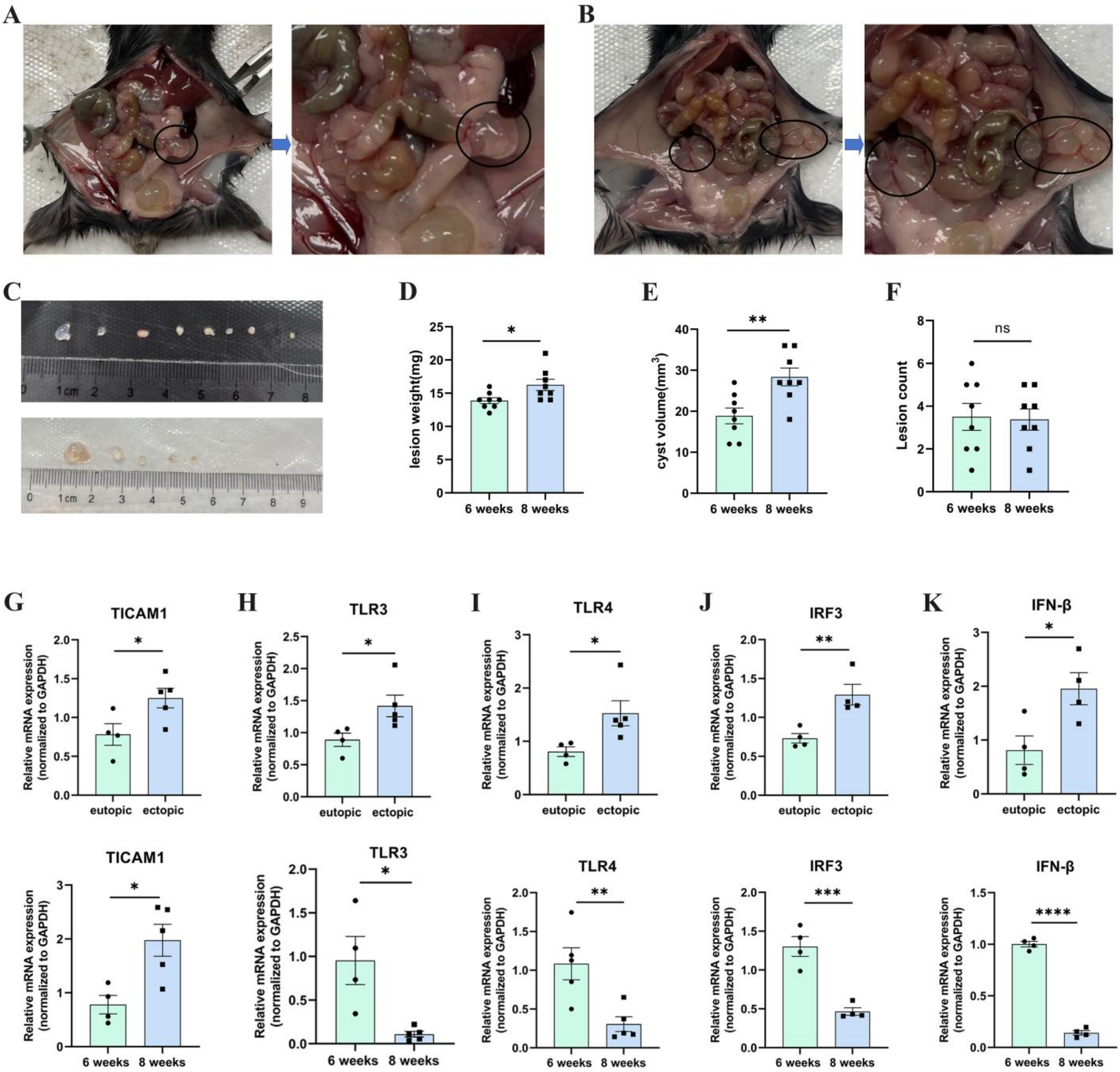

Supplementary Figure S2. Characterization of endometriotic lesions and expression of TICAM1-related signaling molecules in a mouse model. (**A-C**) Macroscopic appearance of endometriotic lesions in mice treated with estrogen for 6 weeks (A, C upper panel) or 8 weeks (B, C lower panel). Lesions are indicated by black circles. (**D-F**) Quantification of lesion weight (D), volume (E), and number (F) in mice treated with estrogen for 6 versus 8 weeks (n = 8). (**G-K**) mRNA expression of TICAM1 (G), TLR3 (H), TLR4 (I), IRF3 (J), and IFN-β (K). Top panels: comparison between eutopic and ectopic endometrium at 8 weeks post-induction. Bottom panels: comparison of ectopic lesions between 6- and 8-week post-induction mice (n = 4–5 for G-I; n = 4 for J-K). Data are presented as mean ± SEM. * $p < 0.05$, ** $p < 0.01$, *** $p < 0.001$, **** $p < 0.0001$; ns, not significant ($p > 0.05$).

Supplementary table S1. RT-qPCR Primer Sequences (Human)

Gene name	Forward primer sequence (5' to 3')	Reverse primer sequence (5' to 3')
GAPDH	GAGAAGGCTGGGGCTCATTT	AGTGATGGCATGGACTGTGG
TICAM1	TGTCAAAGACCAGACGCCACTC	GTAGATGAAGGAGGAGGAGGAGGAG
TLR3	CACCATTCCAGCCTCTTCGT	CAGGGTTTGCGTGTTTCCAG
TLR4	GGTGCCTCCATTTCAGCTCT	ACTGCCAGGTCTGAGCAATC
IRF3	TGGGCCCCCAGATCTGATTA	CACACAGAACCAGAGGGCAT
IFN-β	TCAGAAGCTCCTGTGGCAAT	TAGATGGTCAATGCGGCGTC

Supplementary table S2. RT-qPCR Primer Sequences (Mouse)

Gene name	Forward primer sequence (5' to 3')	Reverse primer sequence (5' to 3')
GAPDH	TGTGAACGGATTTGGCCGTA	ACTGTGCCGTTGAATTTGCC
TICAM1	AGTCCTTGAAGATGAACACAGTAGC	GGCGAGCCACCGTCCAG
TLR3	CTTTCAAACACAAGCATCCAGAATCTC	GAAGGAACCGTTGCCGACATC
TLR4	AGGACTATGTGATGTGACCATTGATG	GATACACCTGCCAGAGACATTGC
IRF3	ATCTCCAACAGCCAGCCTATCTC	AAGTCCATGTCCTCCACCAAGTC
IFN-β	GCGTTCCTGCTGTGCTTCTC	TGTAGGTGAGGTTGATCTTTCCATTC
